# Effects of the Novel Compound DK223 ([1*E*,2*E*-1,2-Bis(6-methoxy-2*H*-chromen-3-yl)methylene]hydrazine) on Migration and Proliferation of Human Keratinocytes and Primary Dermal Fibroblasts

**DOI:** 10.3390/ijms150713091

**Published:** 2014-07-23

**Authors:** Manh Tin Ho, Hyun Sik Kang, Jung Sik Huh, Young Mee Kim, Yoongho Lim, Moonjae Cho

**Affiliations:** 1 Department of Biochemistry, School of Medicine, Jeju National University, Jeju 690-756, Korea; E-Mails: homanhtin@gmail.com (M.T.H.); biochem310@jejunu.ac.kr (Y.M.K.); 2 Department of Pediatrics, School of Medicine, Jeju National University, Jeju 690-756, Korea; E-Mail: medyapsb@naver.com; 3 of Urology, School of Medicine, Jeju National University, Jeju 690-756, Korea; E-Mail: urohjs@jejunu.ac.kr; 4 Division of Bioscience and Biotechnology, Konkuk University, Seoul 143-701, Korea; E-Mail: yoongho@konkuk.ac.kr; 5 Institute of Medical Science, Jeju National University, Jeju 690-756, Korea

**Keywords:** wound healing, keratinocyte migration, fibroblast proliferation, scar formation, skin regeneration

## Abstract

Wound healing plays an important role in protecting the human body from external infection. Cell migration and proliferation of keratinocytes and dermal fibroblasts are essential for proper wound healing. Recently, several studies have demonstrated that secondary compounds produced in plants could affect skin cells migration and proliferation. In this study, we identified a novel compound DK223 ([1*E*,2*E*-1,2-bis(6-methoxy-2*H*-chromen-3-yl)methylene]hydrazine) that concomitantly induced human keratinocyte migration and dermal fibroblast proliferation. We evaluated the regulation of epithelial and mesenchymal protein markers, such as E-cadherin and Vimentin, in human keratinocytes, as well as extracellular matrix (ECM) secretion and metalloproteinase families in dermal fibroblasts. DK223 upregulated keratinocyte migration and significantly increased the epithelial marker E-cadherin in a time-dependent manner. We also found that reactive oxygen species (ROS) increased significantly in keratinocytes after 2 h of DK223 exposure, returning to normal levels after 24 h, which indicated that DK223 had an early shock effect on ROS production. DK223 also stimulated fibroblast proliferation, and induced significant secretion of ECM proteins, such as collagen I, III, and fibronectin. In dermal fibroblasts, DK223 treatment induced TGF-β1, which is involved in a signaling pathway that mediates proliferation. In conclusion, DK223 simultaneously induced both keratinocyte migration via ROS production and fibroblast proliferation via TGF-β1 induction.

## 1. Introduction

As the outer sheath of the human body, the skin absorbs initial damage from injuries or wounds. Disruption of the normal anatomic structure and loss of organ function lead to the repair process known as wound healing. Wound healing integrates different types of dermal cells and immune cells, such as keratinocytes and dermal fibroblasts, to mediate hemostasis, coagulation, and inflammation [1,2]. Wound healing begins with inflammation and immune cell recruitment to protect tissue from foreign invaders, such as bacteria and viruses. The next phase resurfaces the skin by stimulating regeneration and repair through proliferation of fibroblasts, re-epithelialization of keratinocytes, and other mechanisms. The final phase involves scar formation, which can cause concern for some patients when the scar is not aesthetically pleasing. Therefore, several studies have investigated methods to enhance the regeneration process, which is a specific substitution of the damaged tissue, and to reduce skin repair, which causes fibrosis and scar formation [1].

Herbal plants, such as garlic and curcuma, have been used traditionally for wound healing, and it is known that their effects are due to secondary compounds, such as flavonoids, saponins and alkaloids [3]. For example, Lopez-Jornet *et al.* showed that potassium apigenin and other flavonoids present in verbena extract possess powerful anti-inflammatory properties [4]. In addition, Duarte *et al.* demonstrated the use of chamomile extract ointment for stimulating oral mucosa re-epithelialization and formation of collagen fibers after 10 days of treatment [5]. Moreover, these compounds were demonstrated to influence expression of extracellular matrix (ECM) proteins in skin, including collagen and elastin, which are markers of skin wrinkle [6]. Galicka *et al.* reported increased collagen synthesis upon exposure to apigenin glycoside 7-*O*-glucuronide, a flavonoid glycoside, which also promoted procollagen transformation into collagen [7].

Furthermore, wound healing was controlled by matrix metalloproteinases (MMPs), enzymes that degrade the epidermal layer of the ECM, and are related to the cellular migration, invasion and metastasis processes [8]. MMPs, such as MMP1, MMP8, MMP13, play a specific role in mediating collagen degradation to reduce scar formation in wound healing. For example, Dang *et al.* reported upregulation of MMP1 expression to double normal levels, resulting in scarless wounds in fetal rat skin [9]. The role of MMPs in skin remodeling is well known, they are secreted extra-cellularly and regulated ECM protein accumulation in the wound healing process [10]. Notably, it has been reported that bioactive compounds regulate the expression and activity of MMPs. Catanzaro *et al.* demonstrated the effects of a sturgeon-based bioactive compound on the expression of the tumor necrosis factor-alpha, MMPs, and type-10 collagen genes in human chondrocytes [11]. Application of bioactive compounds may offer a potential therapy for controlling the wound healing or skin aging process.

Natural secondary metabolites have been demonstrated to have several potential capabilities in gene expression regulation for skin repair. Based on the knowledge of the original structures, drug development science can be used to synthesize compounds that are conformationally modified to improve their activities [12]. In that way, novel compounds have been synthesized using innovative chemical techniques that expand on the original conformation. In this study, we examined the hypothesis that the novel compound DK223 ([1*E*,2*E*-1,2-bis(6-methoxy-2*H*-chromen-3-yl)methylene]hydrazine) could induce keratinocyte migration, fibroblast proliferation, anti-wrinkle gene expression, and enhance the regeneration of skin, rather than mediate scar formation.

## 2. Results

### 2.1. DK223 Promoted the Migration but not the Proliferation of HaCaT Cells

**Figure 1:**
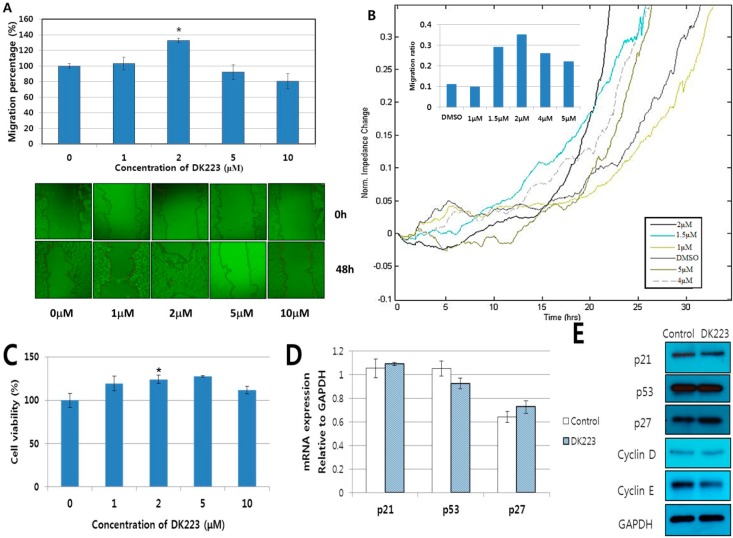
Effect of DK223 on the migration and proliferation of HaCaT cells. (**A**) Cells were seeded at adensity of 3 × 10^4^/mL 24 h before the scratch test. Scratch widths were measured 48 h after DK223 treatment. Data represent the mean width percentage ± SD for at least three replicates, * *p* < 0.05; (**B**) Cells were seeded at adensity of 3 × 10^4^/mL 24 h before treatment. The impedances were measured and analyzed at 24 h after treatment using the ECIS system software. The line data indicate the migration velocity of cells treated with different concentrations of DK223; (**C**) Cells were seeded overnight before treatment with varying concentrations of DK223 for 48 h. Cells were then incubated with MTT for 4 h at 37 °C, and the absorbance was measured at 570nm; (**D**,**E**) The expression of cell cycle-related proteins in HaCaT cells induced by 2 μM DK223 for 24 h. Protein expression was examined by Western blot analysis with the specific antibodies indicated, and mRNA expression was assessed by RT-PCR with the gene-specific primers indicated. A histogram depicting the ImageJ data analysis resultsis shown. Values represent means ± SD of three independent experiments, * *p* < 0.05 compared with the control in all under experiments.

To investigate the ability of DK223 to induce migration of human keratinocytes, we treated HaCaT cells with different concentrations of DK223 in a 48 h scratch wound-healing assay. The results showed that DK223 at 2 μM significantly enhanced migration of HaCaT (167%), whereas other concentrations did not produce marked effects (Figure 1A). In order to confirm these results, we performed the migration assay with the ECIS system using other concentrations of DK223. In this assay, during cell growth, the current is impeded based on the number of cells covering the electrode, which indicates the velocity of cell movement. The results were collected as impedance *versus* time. As expected, 2 μM DK223 significantly enhanced the migration velocity of keratinocytes after 24 h compared with the control (Figure 1B).

We used the MTT assay to assess proliferation of HaCaT cells in response to DK223, and the results demonstrated that there was a significant increase in keratinocyte growth with 2 μM DK223 after 48 h (Figure 1C). Whereas the mRNA and protein expression of p53, p21, p27, cyclin D, and cyclin E did not significantly change in 24 h compared with the untreated cells (Figure 1D,E). These observations indicate that DK223 could induce cell migration, as well as proliferation of human keratinocytes.

### 2.2. DK223 Did not Induced Epithelial–Mesenchymal Transition (EMT) in HaCaT Cells

**Figure 2:**
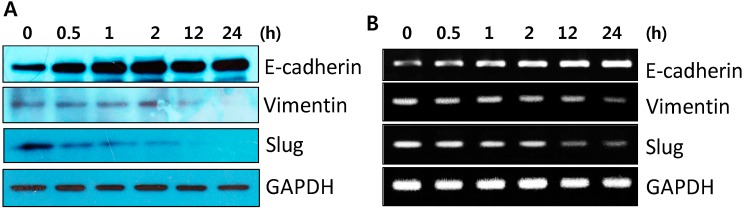
Time-dependent expression of EMT-related genes in HaCaT cellsaftertreatmentwith DK223. (**A**) Protein expression was examined in HaCaT cells treated with 2 μM DK223 for different time periods using Western blot analysis with the specific antibodies indicated (**B**) mRNA expressionwas investigated by RT-PCR with the specific primers indicated.

EMT is critical for wound healing, and tissue regeneration has been shown to induce keratinocyte migration [13]. Thus, we examined the mRNA and protein expression of EMT markers, such as vimentin, E-cadherin, and Slug after DK223 treatment to investigate whether DK223-induced migration affected the EMT process. As shown by Western blot in Figure 2A, two mesenchymal markers, vimentin and Slug, were down-regulated in a time-dependent manner, whereas the adherents junction protein E-cadherin was increased significantly, peaking 24 h after treatment. These results correlated with the changes in mRNA expression measured by RT-PCR (Figure 2B). This suggested that DK223 did not induced EMT, processes in HaCaT cells.

### 2.3. DK223 Induced NADPH Oxidase Expression and ROS Accumulation

**Figure 3:**
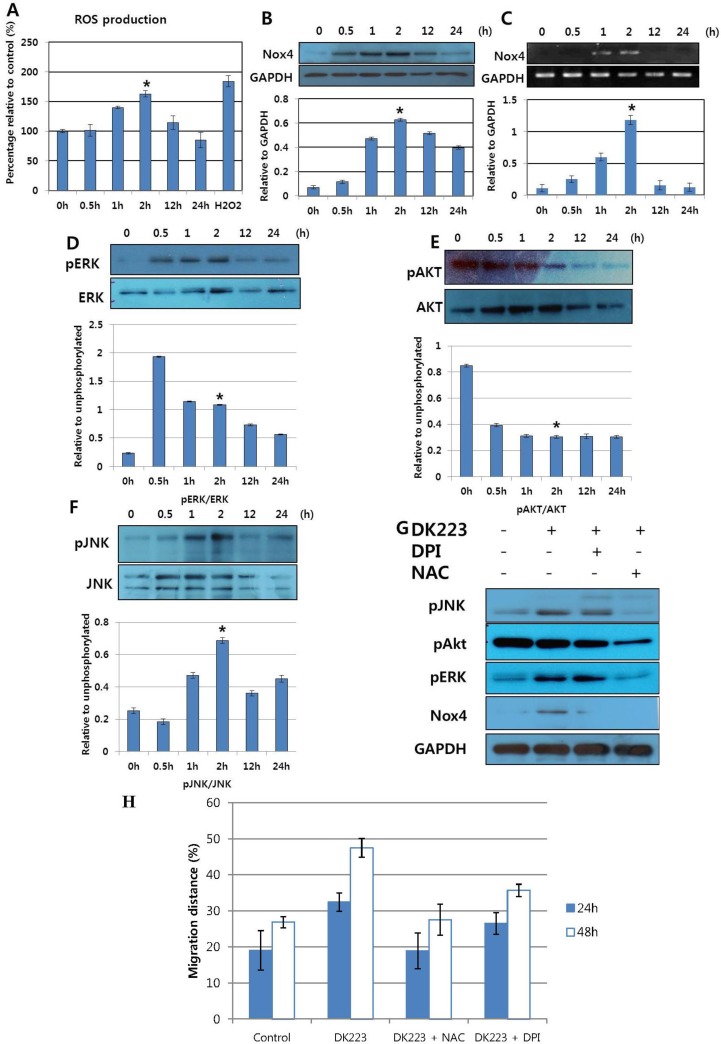
Time-dependent expression of NAPDH oxidase, ROS accumulation and signaling pathways in HaCaT cells after treatment with DK223. (**A**) Time- dependent ROS generation was detected in treated HaCaT cells using the DCF-DA assay. Cells were treated with 2 μM DK223 at different times and then stained with DCF-DA to detect ROS generation, as described previously. H_2_O_2_ was used as a positive control; (**B**,**C**) The expression of NAPDH oxidase 4 was examined by Western blot and RT-PCR, as described previously; (**D**–**F**) Signaling pathways were examined by Western blot with the specific antibodies indicated; (**G**) Cells were pretreated with 10 μM DPI or 20 μM NAC for 2 h before 24 h DK223 incubation. Signaling pathways were examined by Western blot with the specific antibodies indicated; (**H**) Cells were seeded at adensity of 3 × 10^4^/mL 24 h before the scratch test. Scratch widths were measured 48 h after DK223 treatment only or co-treatment with 10 μM DPI or 20 μM NAC. A histogram shows the results of ImageJ data analysis. Values represent means ± SD of three independent experiments, * *p* < 0.05 in all under experiments.

To gain insight into the mechanism of keratinocyte migration induced by DK223, we assessed NADPH oxidase expression and ROS accumulation, as well as the signaling pathways related to cell migration. As demonstrated in Figure 3B,C, NADPH oxidase 4 mRNA and protein expression increased in a time-dependent manner, peaking 2 h after treatment. This correlated with an up-regulation in ROS accumulation, which also peaked at 2 h (Figure 3A), as well as with the effects on cellular signaling pathways. Phosphorylation of AKT was down-regulated at an early time point, while phosphorylation of ERK and JNK increased after 30 min of treatment (Figure 3D–F). These findings suggest a relationship between the early increase in ROS accumulation at 2 h and concomitant changes in intracellular signaling pathways.

### 2.4. DK223 Affected Signaling Pathways and Cell Migration via ROS Accumulation

To explore the mechanism mediating the effects of DK223 on signaling pathways and ROS accumulation, we pretreated cells with the NADPH inhibitor diphenylene iodonium (DPI) or the ROS scavenger *N*-acetylcysteine (NAC) prior to treatment with DK223 for 2 h. As expected, NADPH oxidase 4 was inhibited by DPI and NAC pretreatment. NAC also reduced DK223-induced phosphorylation of JNK and ERK. Interestingly, the activation of AKT, which was down-regulated by DK223 at 2 h, was also attenuated significantly in the presence of NAC and DPI (Figure 3G). These results indicated that DK223 induced the phosphorylation of JNK and ERK at 2 h via ROS accumulation. Interestingly, DK223-induced migration was inhibited significantly by NAC and DPI (Figure 3H), which indicated that the migration induced by DK223 might be regulate by ROS accumulation.

### 2.5. Migration-Related Proteins Are Down-Regulated by DK223 Treatment in HaCaT Cells

**Figure 4:**
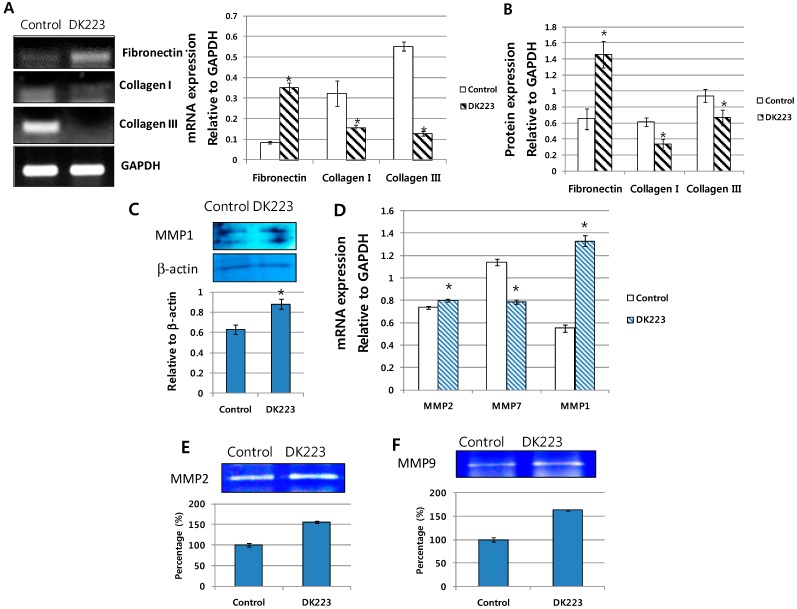
Expression of extracellular matrix proteins and migration-related genes in HaCaT cells treated with DK223. (**A**) mRNA expression was examined in HaCaT cells treated with 2 μM DK223 for 24 h using RT-PCR with the specific primers indicated; (**B**) Cells were treated with 2 μM DK223 in serum-free media. Conditioned media were removed and concentrated using Amicon centrifugation. Proteins in the media were analyzed by Western blot for collagen I, III, and fibronectin using the specific antibodies indicated; (**C**) Proteins in the media was analyzed by Western blot for MMP1 using the specific antibodies indicated; (**D**) mRNA expression was examined in HaCaT cells treated with 2 μM DK223 for 24 h using RT-PCR with the specific primers indicated; (**E**,**F**) Zymograph assay of the conditioned media from HaCaT cells treated with 2 μM DK223 for 24 h. Conditioned media were removed and concentrated using Amicon centrifugation. Proteins in the media were analyzed by a gelatin zymograph, as described previously. A histogram shows the results of ImageJ data analysis. Data are represented as the mean percentage of distance ± SD fromat least three replicates, * *p* < 0.05 in all under experiments.

Because the EMT process did not appear to involve DK223-induced migration, we investigated other migration-related proteins, including the MMPs and collagen I and III in serum-free media which was concentrated by protein filter centrifugation (serum could block the filter). It has been demonstrated recently that these proteins could regulate the migration and proliferation of keratinocytes [14,15]. We observed a significant down-regulation in the mRNA and protein expression of collagen I and III after 24 h of DK223 treatment (Figure 4A–C), while the levels of MMP-1 were increased (Figure 4C) [14]. Thus, high levels of this proteinase might degrade collagen I and III upon DK223 treatment. Moreover, another ECM protein, fibronectin, was highly expressed (Figure 4A–C) with DK223 treatment, which might be associated with the down-regulation of MMP7 (Figure 4D). MMP7 is also known as a matrilysin, since it can use fibronectin as a cleavage substrate [15].

Alternatively, the mRNA expression of two other metalloproteinases, MMP2 and MMP9, also increased slightly compared with the control (Figure 4D), which correlated with results from the zymography assay for gelatinase activity of MMP2 and MMP9 (Figure 4E,F). These results indicate that the effects of DK223 on keratinocyte migration could be mediated by the degradation of collagen via a highly expressed collagenase.

### 2.6. DK223 Ameliorates Proliferation of Human Dermal Fibroblasts

**Figure 5:**
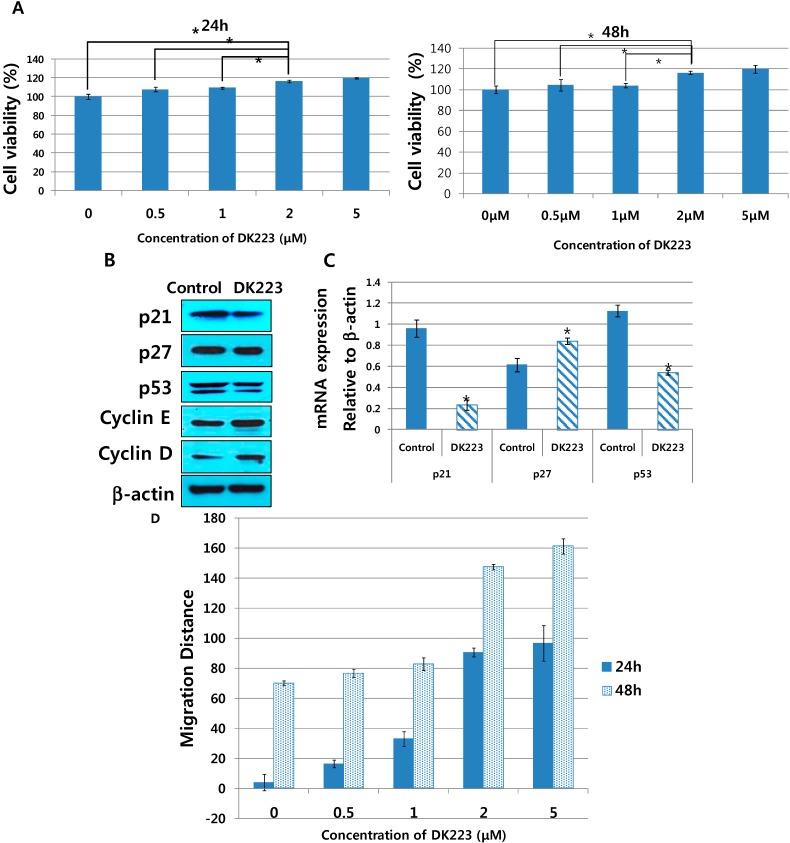
Proliferation and migration of dermal fibroblasts treatedwith DK223 and expression of cell- cycle-related genes in dermal fibroblasts after incubation with DK223. (**A**) Human dermal fibroblasts were seeded at adensity of 3 × 10^4^/mL for 24 and 48 h before treatment. MTT assay was performed as described previously; (**B**,**C**)The expressions of cellcycle-related genes, suchasp21, p53, p27, cyclinE, cyclinD, were examined by Western blot and RT-PCR in 24 h as described previously; (**D**) Cells were seeded at adensity of 3 × 10^4^/mL 24 h before the scratch test. Scratch widths were measured 48 h after DK223 treatment. Ahistogram shows the results of ImageJ data analysis. Data are represented as the mean distance percentages ±SD from at least three replicates, * *p* < 0.05 in all under experiments.

Tissue formation, which is the second phase of wound healing, is characterized by migration and proliferation of different cell types. After the initiation of keratinocyte migration, fibroblasts tend to proliferate [2]. Therefore, we investigated the effects of DK223 on human dermal fibroblasts. We initially examined the proliferation of dermal fibroblasts in response to DK223 for 24 and 48 h using the MTT assay. These results demonstrated that DK223 induced dermal fibroblast proliferation in a dose-dependent manner in both 24 and 48 h (Figure 5A). There was no significant difference between 2 and 5 μM concentrations of DK223; thus, we used 2 μM in subsequent experiments.

To confirm the enhanced proliferation of fibroblasts treated with DK223, we evaluated the mRNA and protein expression of cell cycle-related genes. As shown by Western blot in Figure 5B, the protein levels of p21, p27 and p53 were down-regulated significantly after a 24 h exposure to 2 μM DK223, while cyclin D and cyclin E were increased. These results correlated with changes in mRNA expression measured by RT-PCR (Figure 5C) except for p27 expression, which was increased after DK223 treatment. These data further indicate that the proliferation of fibroblasts increased with 2 μM DK223 after 24 h.

In addition, the migration results showed that DK223 at 2 and 5 μM significantly enhanced migration of fibroblast in both 24 and 48 h, whereas other concentrations did not give high effects (Figure 5D).

### 2.7. Dermal Fibroblasts Promote DK223-Induced ECM Deposition

**Figure 6:**
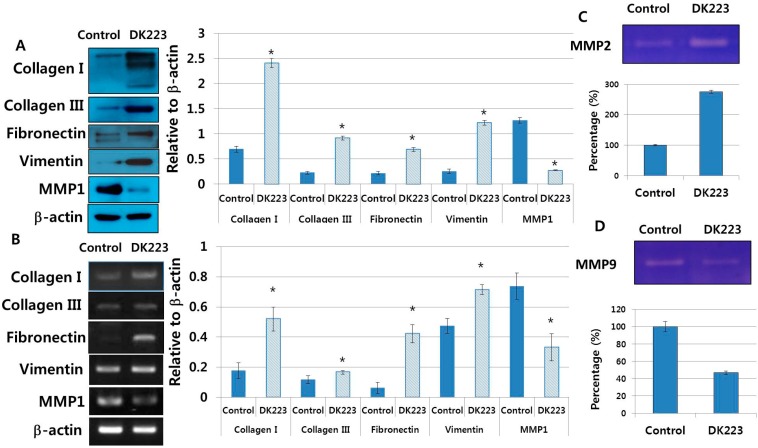
Expression of extracellular matrix proteins and metalloproteinase genes. (**A**) Dermal fibroblasts were treated with 2 μM DK223 in serum-free media. Conditioned media were removed and concentrated using Amicon centrifugation. Proteins in the media were analyzed by Western blot for collagen I, III, fibronectin, MMP1, and vimentin with the specific antibodies indicated; (**B**) mRNA expression was examined in human dermal fibroblast cells treated with 2 μM DK223 for 24 h using RT-PCR and the specific primers indicated; (**C**,**D**) Zymograph assay of conditioned media from dermal fibroblasts treated with 2 μM DK223 for 24 h. Conditioned media were removed and concentrated using Amicon centrifugation. Proteins in the media were analyzed by a gelatin zymograph as describedpreviously.A histogram shows the results of ImageJ data analysis. Data are represented as the mean distance percentages± SD from at least three replicates, * *p* < 0.05.

The ECM contains components essential to wound healing, including collagen I, III, and fibronectin [16]. Therefore, we examined ECM protein secretion from fibroblasts induced by DK223 for 24 h. Western blot analysis revealed high levels of collagen I, III and fibronectin with DK223 treatment (Figure 6A), which correlated with mRNA expression (Figure 6B). Another mesenchymal marker, vimentin, was augmented significantly compared with the control, indicating myofibroblast differentiation and possible migration (Figure 6B). Notably, MMP1, a collagenase protein, was inhibited markedly when treated with DK223 (Figure 6B), which could explain the high levels of collagen I and III in DK223-treated fibroblasts. However, differential effects were seen between MMP2 and MMP9, also known as gelatinase A and gelatinase B. MMP2 was up-regulated in DK223 treatment, while MMP9 was down-regulated (Figure 6C,D). Thus, correlating the expression of these MMPs with our results requires further investigation.

### 2.8. DK223 Induces TGF-β11 Secretion in Fibroblasts

**Figure 7:**
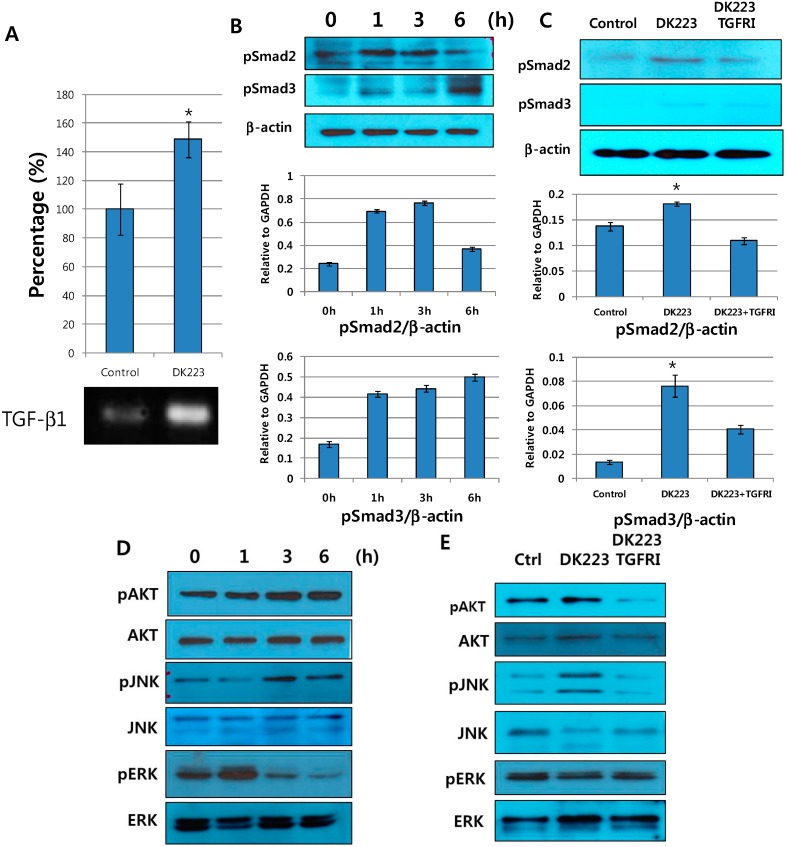
The effect of DK223 on TGF-β1 expression and on dermal fibroblast signaling pathways in response to a TGF-β1 receptor inhibitor. (**A**) Dermal fibroblasts were treated with 2 μM DK223 in serum-free media. TGF-β1 mRNA expression was examined in fibroblast cells treated with 2 μM DK223 for 48 h using RT-PCR with specific primers; (**B**,**D**) Dermal fibroblasts were treated with 2 μM DK223 in serum-free medium for different time periods; (C,E) Dermal fibroblasts were pretreated with 10 μM TGF-β1 receptor inhibitor (TGFRI)—LY2157299 before incubating with DK223 for 6 h. Signaling pathways were examined by Western blot with the specific antibodies indicated. A histogram shows the results of ImageJ data analysis. Values represent the means ± SD of three independent experiments, * *p* < 0.05 compared with the control in all under experiments.

Fibroblasts have a key role in wound healing because they secrete growth factors and cytokines, such as TGF-β1, connective transforming growth factor (CTGF), IL-6 and IL-8 [17]. We investigated the production of TGF-β1 in DK223-treated fibroblasts to determine whether proliferation could be correlated with collagen deposition in fibroblasts. We found that DK223 significantly increased TGF-β1 mRNA expression (Figure 7A).

It has been suggested that TGF-β1 might promote fibroblast proliferation [18]. Thus, we investigated the correlations between DK223-induced TGF-β1 cellular changes in fibroblasts by examining the activation of various signaling pathways. The results demonstrated that the phosphorylation of AKT, a potential oncogene, was elevated in a time-dependent manner (Figure 7D). In addition, we observed early activation of pSmad2 at 1 h and increased phosphorylation of pSmad3 (Figure 7B) accompanied by the significant activation of JNK at 6 h (Figure 7D). These events indicate that DK223 activates the JNK/pSmad3L/c-Myc oncogenic pathway that mediates increased proliferation of fibroblasts. Interestingly, ERK phosphorylation was down-regulated below basal levels after an initial early increase at 1 h (Figure 7D). This could be the mechanism underlying the anti-proliferative response of the Ras/Raf/MEK/ERK signaling pathway that was previously reported in primary human fibroblasts [19,20]. Taken together, these results suggest that TGF-β1 signaling pathways mediate fibroblast proliferation in response to DK223.

To confirm the involvement of TGF-β1, we treated cells with a TGF-β1 receptor inhibitor (TGFRI), which significantly inhibited pSmad2, pSmad3, JNK, and Akt phosphorylation (Figure 7C,E). Furthermore, TGFRI pretreatment restored ERK phosphorylation to normal levels (Figure 7E). In summary, our results indicate that DK223 modified cellular signaling pathways by elevating TGF-β1 secretion.

## 3. Discussion

The wound repair process protects the human body from infections and fluid loss; yet, it results in scar formation. In this study, we investigated the effects of DK223 on wound healing processes with the expectation that DK223 could induce skin regeneration.

Skin regeneration is characterized by a re-epithelialization process that covers the wound surface and stimulates re-angiogenesis [21]. After the wound has been blocked by a blood clot, fibroblasts begin secreting ECM proteins, such as collagen III and fibronectin. These proteins reorganize the base structure under the blood clot to aid in keratinocyte migration [1]. We have studied this phenomenon by treating human keratinocytes with different concentrations of DK223 *in vitro*. While DK223 did not promote keratinocyte cell growth, migration was enhanced significantly. Mesenchymal markers, such as Vimentin and Slug, were down-regulated in a time-dependent manner, whereas the epithelial marker E-cadherin was increased markedly. This indicates that keratinocytes did not induce the EMT process and that the cells were bound together more tightly as migration increased. This phenomenon was compatible with our previous report of increased keratinocyte migration and higher levels of junction and adherent proteins, such as E-cadherin [22]. This was also reported by Li *et al.*, who showed that a whole sheet of epithelial cells could migrate while maintaining tight cell-cell adhesion, a phenomenon they called “collective migration” [23]. As shown in Figure 3, DK223 significantly up-regulated E-cadherin, which plays an important role in re-epithelialization since adherent junctions prevent the epithelial barrier from further damage after wounding [23]. This indicates that DK223 was able to induce collective migration to protect the epithelial barrier from wounds.

In a recent study, Kim *et al.* demonstrated that HaCaT keratinocyte migration was induced by the EGF-like ligand neuregulin, which was activated via Rac1 and Nox-driven ROS accumulation [24]. In our study, DK223 also significantly increased HaCaT cell migration via intracellular signaling pathways. Our results demonstrated that ROS accumulation peaked at 2 h, along with signaling proteins, such as AKT and ERK. In recent studies, AKT2 was shown to induce cell migration [25] and ERK/MAPK phosphorylation might be activated by ROS [26,27]. In addition, ERK/MAPK phosphorylation ameliorated keratinocyte migration [28]. In light of these results, we propose that DK223 induces keratinocyte migration by modulating intracellular signaling pathways via ROS accumulation.

Dermal fibroblasts are the primary ECM-producing cell type in the skin [29]. Once the wound has formed a blood clot via fibrin, dermal fibroblasts proliferate and infiltrate under the wound [16]. These cells secrete collagen and fibronectin, which replace the clot fibrin to accelerate wound closure [16]. Nevertheless, ECM accumulation in instances of excessive healing may cause unaesthetic scars [2]. In this study, DK223 induced keratinocyte migration and promoted the fibroblast-to-myofibroblast transition to help the wound heal more rapidly [30], with the evidence that vimentin was upregulated in fibroblasts, whereas it modulated ECM production in keratinocytes and fibroblasts. Furthermore, DK223 treatment increased fibronectin expression in both fibroblasts and keratinocytes. It is well known that fibronectin, which is normally produced in the first stage of wound healing, acts as the scaffold for collagen deposition and assists in cell migration to promote the wound healing process [31,32]. Collagens I and III are the main products of the wound healing process. Production of these collagens is necessary for wound healing; however, collagen deposition can promote scar formation. Thus, collagen production must be controlled for ideal wound healing. The DK223 effect resulted in increased collagen in fibroblasts, whereas a reduction in keratinocytes was seen. Correspondingly, levels of the collagenase MMP1 were also increased in keratinocytes and reduced in fibroblasts. These observations suggest a regulatory role for DK223 in maintaining the balance of ECM production in skin repair. Similarly, Xie *et al.* reported that basic fibroblast growth factor (bFGF) improved wound healing by regulating the balance of ECM synthesis and degradation [33]. These simultaneous effects balance ECM production in the wound healing process and might prevent keloid scar formation. Thus, DK223 differentially modulates MMPs and ECM production in keratinocytes and fibroblasts to regulate individual cell functions during wound healing.

According to our data, DK223 affects both keratinocyte migration and fibroblast proliferation to accelerate the wound healing process. It has been previously demonstrated that other novel compounds also exhibit similar effects. Lee *et al.* reported that heat-processed ginseng (sun ginseng) protected against UVB-induced damage in keratinocytes and fibroblasts by restoring Bcl-2 and Bcl-xL mRNA levels in keratinocytes to reduce UVB-induced apoptosis, and promote procollagen production in dermal fibroblasts by inhibiting MMP-1 gene expression [34]. Werner *et al.* also reported that the keratinocyte-fibroblast interaction is also important for wound healing as keratinocytes modulate fibroblast to myofibroblast differentiation by secreting the growth factor-like TGF-β1, which increases production of myofibroblasts in ECM [35]. Moreover, Singh *et al.* suggested an alternative approach for wound healing by focusing on the effects of traditional medicines on keratinocytes and fibroblasts. Their bioactive compound increased both types of skin cells and reduced free-radical production to support the wound closure process [36].

TGF-β1 can participate in the wound healing process by inducing fibroblast migration and proliferation [37], activating the MMP family [38] and stimulating re-epithelialization [37]. It has been demonstrated that TGF-β1 expression was elevated significantly by excessive wound healing, leading to keloid scarring [39,40]. In our study, DK223 slightly increased TGF-β1 expression in fibroblasts, indicating that it did not promote scarring.

## 4. Materials and Methods

### 4.1. Reagent

**Figure 8:**
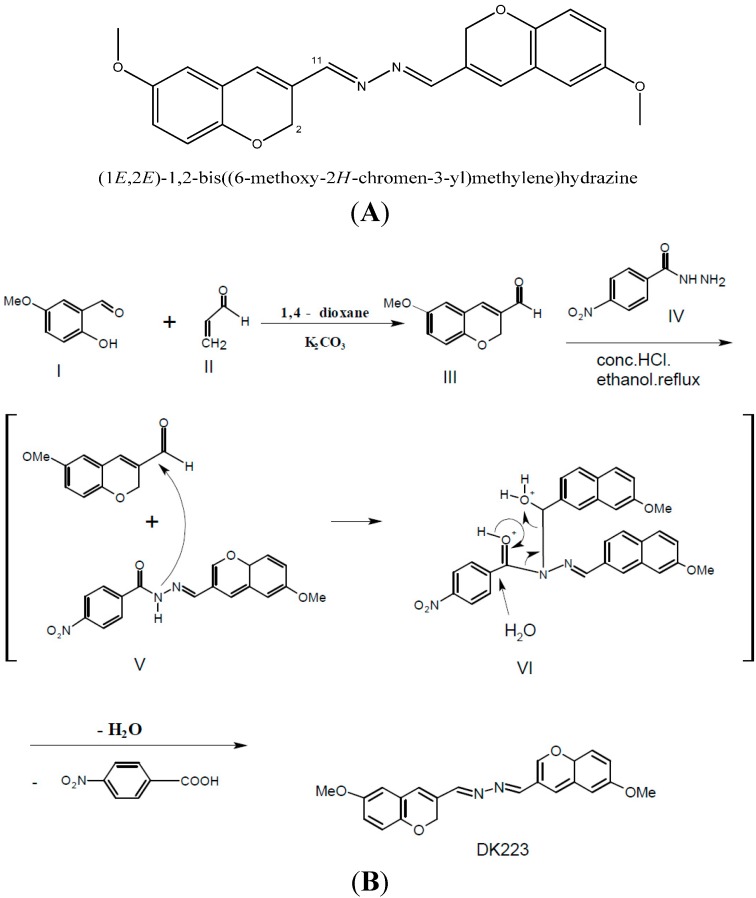
(**A**) Chemical structure of DK223; (**B**) Method of DK223 synthesis, as described in the Materials and Methods section.

The DK223 (Figure 1A) compound was synthesized and kindly provided by Youngho Lim in the Division of Bioscience and Biotechnology, Konkuk University, Seoul, Korea. K_2_CO_3_ (5 mmol, 692 mg) was dissolved in a solution of 2-hydroxy-5-methoxybenzaldehyde (**I**, 5 mmol, 760 mg) and acrolein (**II**, 7 mmol, 0.5 mL) in 1,4-dioxane (50 mL) and the reaction mixture was refluxed for 6 h. The solvent was evaporated and residue was extracted with diethyl ether (100 mL) and water (50 mL). Combined organic layers were dried over MgSO_4_. Filtration and evaporation produced aldehyde (**III**, 86%, m.p. 48–50 °C), which was used for next step without further purification. *p*-Nitroacylhydrazine (**IV**, 2 mmol, 334 mg) and 6-methoxy-2*H*-chromene-3-carbaldehyde (III, 3 mmol, 570 mg) were dissolved in ethanol (30 mL) and a catalytic volume of conc. HCl was added. The resulting mixture was refluxed for 2 h. The reaction formed intermediates **V** and **VI**, which eventually produced DK233. A similar reaction has been reported previously [41]. After cooling to room temperature, the resulting precipitate was filtered and washed with ethanol. Recrystallization of crude solid resulted in pure DK223 (355 mg, 50%, m.p. 246–250 °C; (Figure 8B)).

### 4.2. Cell Culture

In this study, we used the spontaneously immortalized keratinocyte cell line (HaCaT) and human dermal fibroblasts. HaCaT cells were cultured in RPMI medium (GIBCO, Grand Island, NY, USA) supplemented with 10% fetal bovine serum (FBS; GIBCO) and 1% penicillin/streptomycin (PAA Laboratories GmBH, Strasse, Austria). Human dermal fibroblast cells were cultured in DMEM media supplemented with 10% FBS (GIBCO) and 1% penicillin/streptomycin (PAA). Cells were incubated in a humidified atmosphere at 37 °C with 5% CO_2_.

### 4.3. Scratch Wound Healing Assay

HaCaT cells were seeded in a 48-well plate before a 24 h wound healing scratch assay [42]. The scratch wound was made using a sterile 200 µL pipette tip to scratch a line across the bottom of the culture dish. Culture media was then removed and replaced with fresh media supplemented with DK223 or dimethyl sulfoxide (DMSO) (Amresco, Solon, OH, USA) as a control. Photographs were taken at 4× magnification using an OLYMPUS IX70 microscope equipped with a digital camera before treatment and 48 h after treatment. The width of the scratch was measured by the distance from the both edge of scratch by ImageJ software (National Institutes of Health, Bethesda, MD, USA). Each scratch was measured 60 times across the span of wound scratch and calculated the average to compare the migration after 24 or 48 h. The experiments were repeated 3 dependent times.

### 4.4. Migration Assay with the Electric Cell-Substrate Impedance Sensing (ECIS) System

ECIS^®^ (Electric Cell-substrate Impedance Sensing) is a real-time, label-free, impedance-based method of assessing the activities of cells grown in tissue culture. When cells are added to the ECIS Arrays and attached to the electrodes, they act as insulators and increase the impedance. As cells grow and cover the electrodes, the current is impeded in a manner related to the number of cells covering the electrode, the morphology of the cells, and the nature of the cell attachment. When cells are stimulated to change their function, the accompanying changes in cell morphology alter the impedance. Data are generated as impedance *versus* time.

Electrode plates (eight wells, Applied Biophysics, Troy, NY, USA) were induced with an electrode-stabilizing solution provided by the manufacturer 10 min prior to seeding with HaCaT cells overnight. Cells were then treated with different concentrations of DK223. After 24 h, the electrode plates were placed in the ECIS apparatus in the incubator for migration analysis using the ECIS system software, according to the manufacturer’s instructions (Applied Biophysics, Troy, NY, USA).

### 4.5. MTT Assay

Cells were seeded in a 96-well plate at a density of 3 × 10^4^/mL. HaCaT cells were treated with DK223 or DMSO for 24 h, while dermal fibroblasts were treated for 48 h. MTT solution 5 mg/mL (Amresco, OH, USA) was added to each well and incubated at 37 °C for 4 h. The medium was then gently removed and replaced with 150 μL DMSO, followed by shaking for 30 min to dissolve the precipitate, and the absorbance was measured at 570 nm.

### 4.6. Western Blot

Cells were treated with DK223 or DMSO in a time- and dose-dependent manner. Cells lysate proteins were then harvested using a cell scraper and lysed in RIPA buffer. The protein concentration was determined using the bicinchoninic (BCA) assay (Thermo Scientific, Rockford, IL, USA). The protein in conditioned media (serum free media after treated cell with DK223) was concentrated by Amicon (Milipore, Darmstadt, Germany) centrifuge following manufacture instruction. The protein in media after centrifugation was determined the concentration using the Bardford assay (Bio-Rad, Hercules, CA USA). The proteins were separated by SDS-PAGE, followed by Western blot using the appropriate antibodies.

All antibodies for Western blot were purchased commercially and prepared according to the manufacturer’s protocol. p21 (2947), AKT (9272), phosphorylated AKT (9271S), ERK (4695), Slug (9585S), and GAPDH (2118) were obtained from Cell Signaling (Danvers, MA, USA). E-cadherin (610181) was purchased from BD Science Transduction (San Jose, CA, USA). Cyclin E (sc-247), cyclin D1 (sc-246), p53 (sc-126), phosphorylated ERK (sc-7383), collagen I (sc-25974), collagen III (sc-28888), and fibronectin (sc-9068) were purchased from Santa Cruz Biotechnologies (Santa Cruz, Dallas, TX, USA). MMP1 (444209) was purchased from Calbiochem (Hessen, Darmstadt, Germany). We used anti-mouse (PI-2000, Vector Labs Inc., Burlingame, CA, USA), anti-rabbit (PI-1000, Vector Labs), and anti-goat (AP-107P, Millipore, Billerica, MA, USA) secondary antibodies. Differences were confirmed using ImageJ software to determine the relative ratio of changes in the target protein levels to those of the DMSO control.

### 4.7. RT-PCR

Total RNA from treated cells was extracted using TRIzol reagent (Invitrogen, Grand Island, NY, USA). Equal amounts of total RNA in each treatment group were used to synthesize cDNA using a reverse transcriptase kit (Promega, Seoul, Korea). The resulting cDNA was used for RT-PCR using the G-Taq kit (Cosmo Genetech, Seoul, Korea) based on the manufacturer’s protocols.

RT-PCR was performed using the following gene-specific primers designed using the Blast Primer website: GAPDH, forward primer 5'-GAAGGTGAAGGTCGGAGTC-3', reverse primer 5'-GAAGATGGTGATGGATTTC-3'; p53, forward primer 5'-ACACGCTTCCCTGGATTGG-3', reverse primer 5'-CTGGCATTCTGGGAGCTTCA-3'; p21, forward primer 5'-GTCAGTTCCTTGAGCCG-3', reverse primer 5'-GAAGGTAGAGCTTGGGCAGG-3'; and MMP1, forward primer 5'-AGGGGAGATCATCGGGAC-3', reverse primer 5'-GGCTGGACAGGATTTTGG-3'; MMP2, forward primer 5'-AACACCTTCTATGGCTGCCC-3', reverse primer 5'-ACGAGCAAAGGCATCACCA-3'; MMP7, forward primer 5'-TACAGTGGGAACAGGCTAGG-3', reverse primer 5'-GGCACTCCACATCTGGGC-3'. ImageJ software was used to analyze the results and the relative ratio of changes in the target gene to those of the DMSO control.

### 4.8. MMP Zymograph Assay

An MMP zymograph assay was performed according to the method described by Gogly *et al.* and modified with use of 8% sodium dodecyl sulfate (SDS) gels mixed with gelatin (0.01 mg/mL). The SDS in the gels was removed by two 30 min incubations in 200 mL 2.5% Triton X-100 at 4 °C, and the gel slabs were incubated at 37 °C overnight in incubation buffer. The gels were subsequently fixed and stained for 1 h in 0.05% Coomassie Blue R-250. The molecular weight protein marker was clearly visible as light blue bands against the blue background. Gelatinase activity was detected as clear zones of negative staining against the blue background. The gels were scanned as permanent records of the results [43].

### 4.9. ROS Generation Analysis

Accumulation of ROS was measured based on ROS-dependent oxidation of the oxidation-sensitive fluorescent probe 2'7'-dichlorofluorescin diacetate (DCFH-DA) to DCF [44]. Cells were seeded in a 96-well plate (3 × 10^4^ cells/mL) for 24 h before flavonoid treatment and then treated with 2 µM DK223 for 0, 0.5, 1, 2, 12, and 24 h. H_2_O_2_ was used as a positive control. The cells were washed with PBS before removing medium, 200 μL of DCFH-DA (100 μM in PBS containing 1% FBS) were added, and the cells were incubated for 30 min at 37 °C in the dark. Intracellular ROS accumulation was measured using DCFH-DA with a DCF-DA microplate assay. A spectrofluorometer (SPECTRAFLUOR, Tecan, Männedorf, Switzerland) was used to assess ROS generation by measuring the fluorescence intensities from 10,000 cells/well at an excitation wavelength of 485 nm and an emission wavelength of 530 nm.

### 4.10. Statistics

The results were expressed as means ± standard deviation (S.D.). Group comparisons were performed using the SPSS v.16.0 software (SPSS Inc., Chicago, IL, USA) with one-way analysis (ANOVA) and Student’s *t*-test. *p* < 0.05 was considered to indicate statistical significance. All experiments were repeated at least in triplicate.

## 5. Conclusions

In conclusion, the novel compound DK223 has potent capabilities in wound healing and skin regeneration. It induced keratinocyte migration and fibroblast proliferation, while maintaining balance among ECM proteins, such as collagen I, III, and fibronectin. Taken together, DK223 potentially aids wound healing and attenuates keloid scar development.
